# Primary leiomyosarcoma of the inferior vena cava in a pediatric case: a case report and literature review

**DOI:** 10.1186/s40792-023-01630-x

**Published:** 2023-04-06

**Authors:** Kazuki Yoshizawa, Yasunari Ohno, Takashi Kurata, Yuki Takagi, Tomoko Kasai, Momoko Takizawa, Yuji Soejima

**Affiliations:** 1 Division of Gastroenterological, Hepato-Biliary-Pancreatic, Transplantation and Pediatric Surgery, Department of Surgery, Shinshu University School of Medicine, 3-1-1 Asahi, Matsumoto, Nagano, Japan; 2 Department of Hematology/Oncology, Nagano Children’s Hospital, 3100 Toyoshina, Azumino, Nagano, Japan; 3 Division of Cardiovascular Surgery, Department of Surgery, Shinshu University School of Medicine, 3-1-1 Asahi, Matsumoto, Nagano, Japan; 4 Department of Surgery, Nagano Children’s Hospital, 3100 Toyoshina, Azumino, Nagano, Japan; 5 Department of Laboratory Medicine, Shinshu University Hospital, 3-1-1 Asahi, Matsumoto, Nagano, Japan

**Keywords:** Leiomyosarcoma, Soft tissue tumor, Childhood, Inferior vena cava, Metastasis, Hepatectomy, Trabectedin, Eribulin, Pazopanib

## Abstract

**Background:**

Leiomyosarcoma is classified as a soft tissue sarcoma. In adults, leiomyosarcoma is the most common malignancy affecting the vascular system; however, vascular leiomyosarcoma in children is extremely rare as most pediatric soft tissue tumors are rhabdomyosarcomas. The survival rate is very low, and incomplete resection is a poor prognostic factor. There is also a high rate of distant recurrence, with the lungs and liver being the most common sites of metastasis. There is no established effective chemotherapy, and complete surgical resection is the only potentially curative treatment for leiomyosarcoma.

**Case presentation:**

A 15-year-old female patient with no significant medical history presented with severe upper abdominal pain and was admitted. Abdominal contrast-enhanced computed tomography and magnetic resonance imaging showed a large retroperitoneal tumor protruding into the lumen of the inferior vena cava behind the liver and multiple small nodules, and metastasis to the liver was suspected. The tumor was 6 × 4 × 5 cm in diameter, located just behind the hepatic hilar structures, and was suspected to infiltrate into the right portal vein. The tumor was diagnosed as a leiomyosarcoma through an open tumor biopsy. As the multiple liver metastases were located only in the right lobe of the liver on imaging, we performed tumor resection with right hepatectomy and replacement of the inferior vena cava (IVC). The postoperative course was uneventful; however, on postoperative day 51, distant metastatic recurrences were found in the remaining liver and right lung. The patient was immediately started on chemotherapy and trabectedin proved to be the most effective drug in the treatment regimen; however, severe side effects, such as hepatotoxicity, prevented timely administration, and the patient passed away 19 months after surgery.

**Conclusions:**

IVC resection and reconstruction combined with right hepatectomy were able to be safely performed even in a pediatric case. To improve the prognosis of leiomyosarcoma with multiple metastases, an effective treatment strategy combining surgical treatment and chemotherapy, including molecularly targeted drugs, should be established as early as possible.

## Background

Leiomyosarcoma is classified as a soft tissue sarcoma. Sarcomas are malignant tumors that arise from primitive mesenchymal tissue. Pediatric soft tissue sarcomas account for 7% of all pediatric tumors, the majority of which are rhabdomyosarcomas, and leiomyosarcomas are very rare, accounting for 1.8% of all soft tissue sarcomas [1]. There are several reports of pediatric cases of mandibular, bronchopulmonary, ovarian, and brain primary tumors; however, to the best of our knowledge, there are only two previous reports describing a pediatric primary vascular tumor such as this one [2–7]. Therefore, data on treatment and prognosis are limited to adults.

In adults, however, leiomyosarcoma is the most common malignancy affecting the vascular system; it mostly occurs in veins rather than arteries and in more than 50% of cases, it originates in the inferior vena cava (IVC) [8, 9]. According to various small, single-center, retrospective studies published to date, survival rates are low, ranging from 31% to 62%, with incomplete resection being a poor prognostic factor [9–18]. In contrast to other sarcomas arising in the retroperitoneum, where local recurrence is common, vascular leiomyosarcoma has a high rate of distant recurrence, with the lungs and liver being the most common metastatic sites [9–19]. As there is no established effective chemotherapy at present, complete surgical resection remains the only potentially curative treatment for leiomyosarcoma.

Herein, we report a case of a 15-year-old girl with primary IVC leiomyosarcoma. Despite the fact that she had already developed multiple liver metastases at the time of diagnosis, we performed partial IVC resection along with a combined right hepatectomy and replaced the IVC with a vascular prosthesis. We present our experience along with a literature review.

## Case presentation

**Fig. 1:**
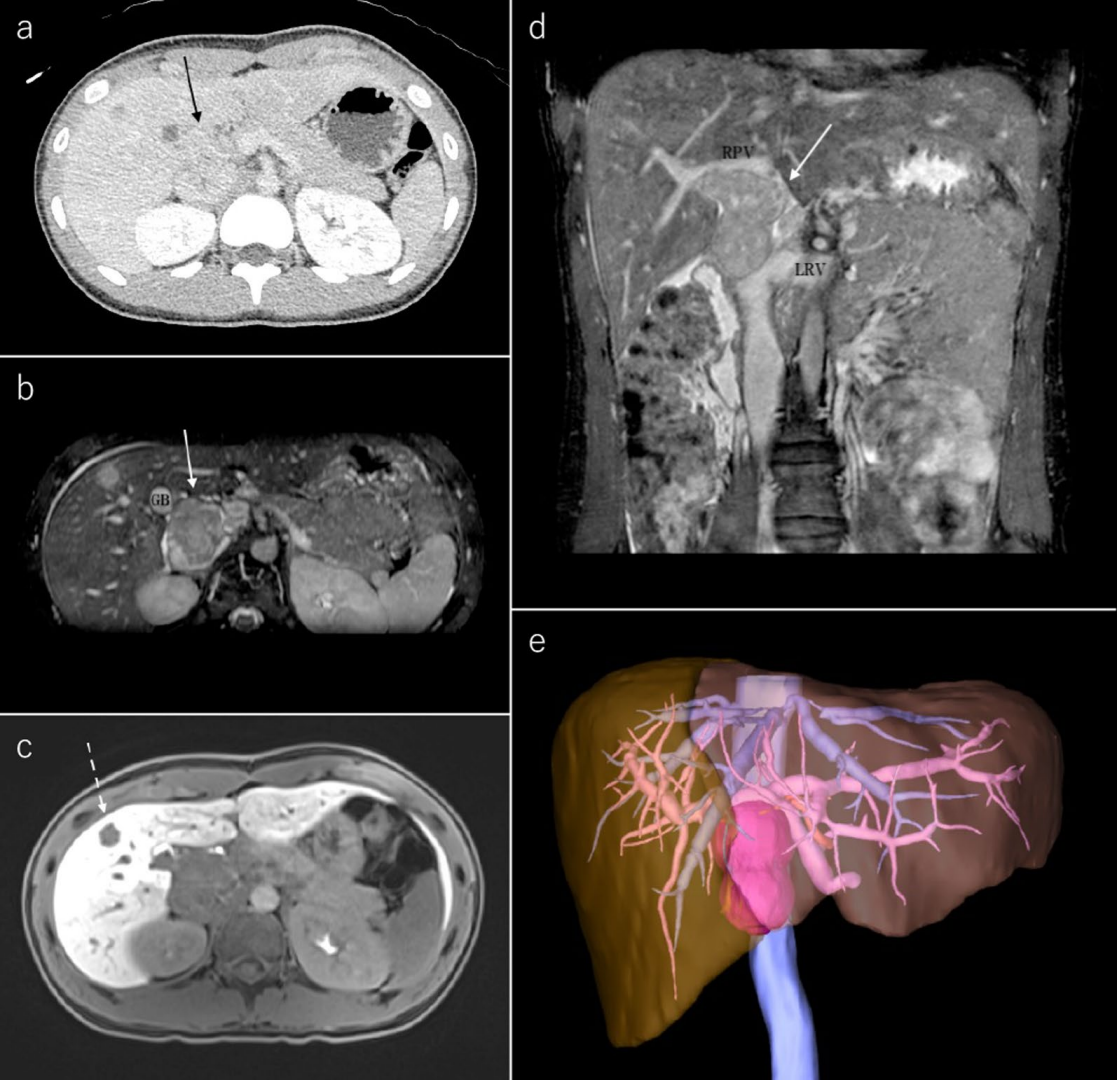
**a**–**d** Contrast-enhanced abdominal CT (**a**) and MRI (**b**–**d**) showed a 6 × 4 × 5 cm mass (solid arrow) protruding into the lumen of the IVC. The mass was located slightly superior to the renal vein confluence, ventrally compressing the hepatic hilum, and the right portal vein was stenotic. The MRI showed areas of decreased Gd-EOB–DTPA uptake at S1 (5 mm × 1), S5 (15 mm × 1, dotted arrow), and S6 (5 mm × 1) in the hepatocellular phase (**c**) **e** Three-dimensional reconstructed image. *GB* gall bladder, *RPV* right portal vein, *LRV* left renal vein, *CT* computed tomography, *MRI* magnetic resonance imaging

A 15-year-old previously healthy girl, of height 151.1 cm and weight 46.5 kg, reported severe upper abdominal pain and a retroperitoneal tumor was found on ultrasound examination. Subsequent abdominal contrast-enhanced computed tomography (CT) and magnetic resonance imaging (MRI) showed a large retroperitoneal tumor protruding into the lumen of the IVC behind the liver. The tumor was 6 × 4 × 5 cm in size, located just behind the hepatic hilar structures, and was suspected to infiltrate the right portal vein (Fig. 1). An open tumor biopsy was performed and the tumor was diagnosed as leiomyosarcoma. All liver nodules were detected on the hepatobiliary phase of contrast-enhanced MRI with Gd-EOB–DTPA as hypointense nodules, suggesting multiple liver metastases (Fig. 1) Lymph node metastases and distant metastases, except for the liver, were not observed on positron emission tomography (PET). Liver function was Child–Pugh score A, and even though the patient had multiple liver metastases (located in the right lobe of the liver on images), we thought that curative resection could be performed. Since there was no reported effective preoperative chemotherapy treatment for leiomyosarcoma, surgical treatment was selected as the preferred management option. This decision was determined through a shared decision-making process with a pediatric oncologist.

As a result, we performed combined tumor resection with a right hepatectomy and replacement of the IVC. The tumor was adherent to the liver, gallbladder, and duodenum, but no ascites or peritoneal dissemination was observed. The IVC on the superior and inferior side of the tumor and the right and left renal veins were taped circumferentially. The gallbladder was resected together with the tumor due to suspected tumor invasion but the duodenum and hilar region were easily detached from the tumor and invasion was negative. The IVC was dissected 2 cm from the tumor on the superior side and just above the bifurcation of the right and left renal veins on the inferior side, and the tumor was removed along with the IVC.

**Fig. 2:**
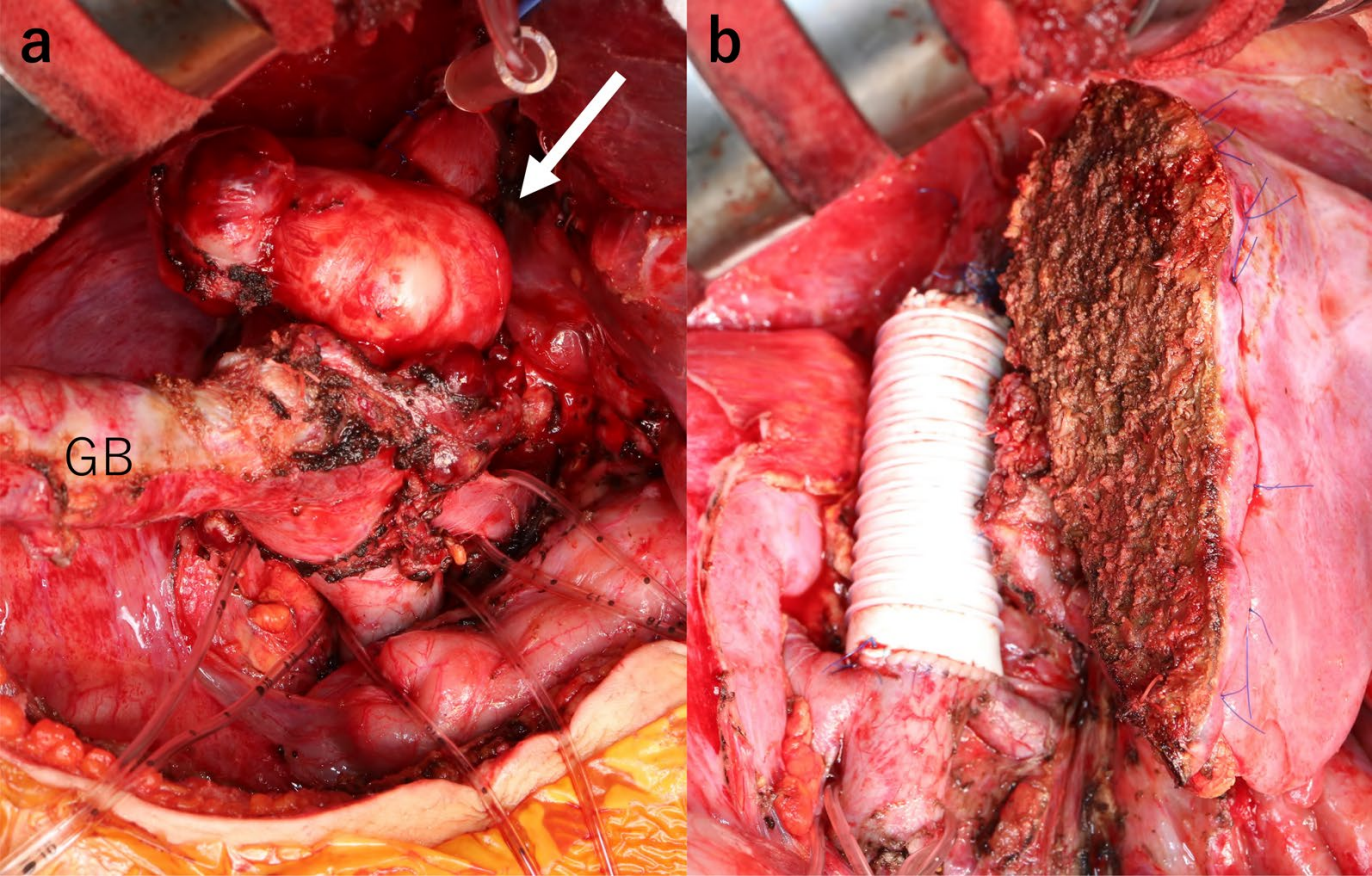
**a** Hard elastic mass (arrow; 6 × 5 cm in size) found in the IVC on the superior side of the renal vein confluence. **b** Defect of the IVC was reconstructed using a 6 cm Gore-Tex® e-PTFE graft (φ20 mm). *GB* gall bladder, *IVC* inferior vena cava

The tumor was 6 × 5 cm in size, elastic and hard. The defect of the IVC was reconstructed using a 6 cm Gore-Tex® expanded polytetrafluoroethylene (e-PTFE) graft (φ20 mm) (Fig. 2). Intraoperative ultrasonography revealed a 15 mm nodular lesion in the liver S5 and 5 mm nodular lesions in S6 and S1 (right side); there were no obvious nodules on the left side of the liver or in S1 (left side). Liver transection was performed, and the right lobe and part of S1 were removed. The operation time was 9 h 45 min, the total ischemic time was 35 min, and the blood loss was 700 ml.

**Fig. 3:**
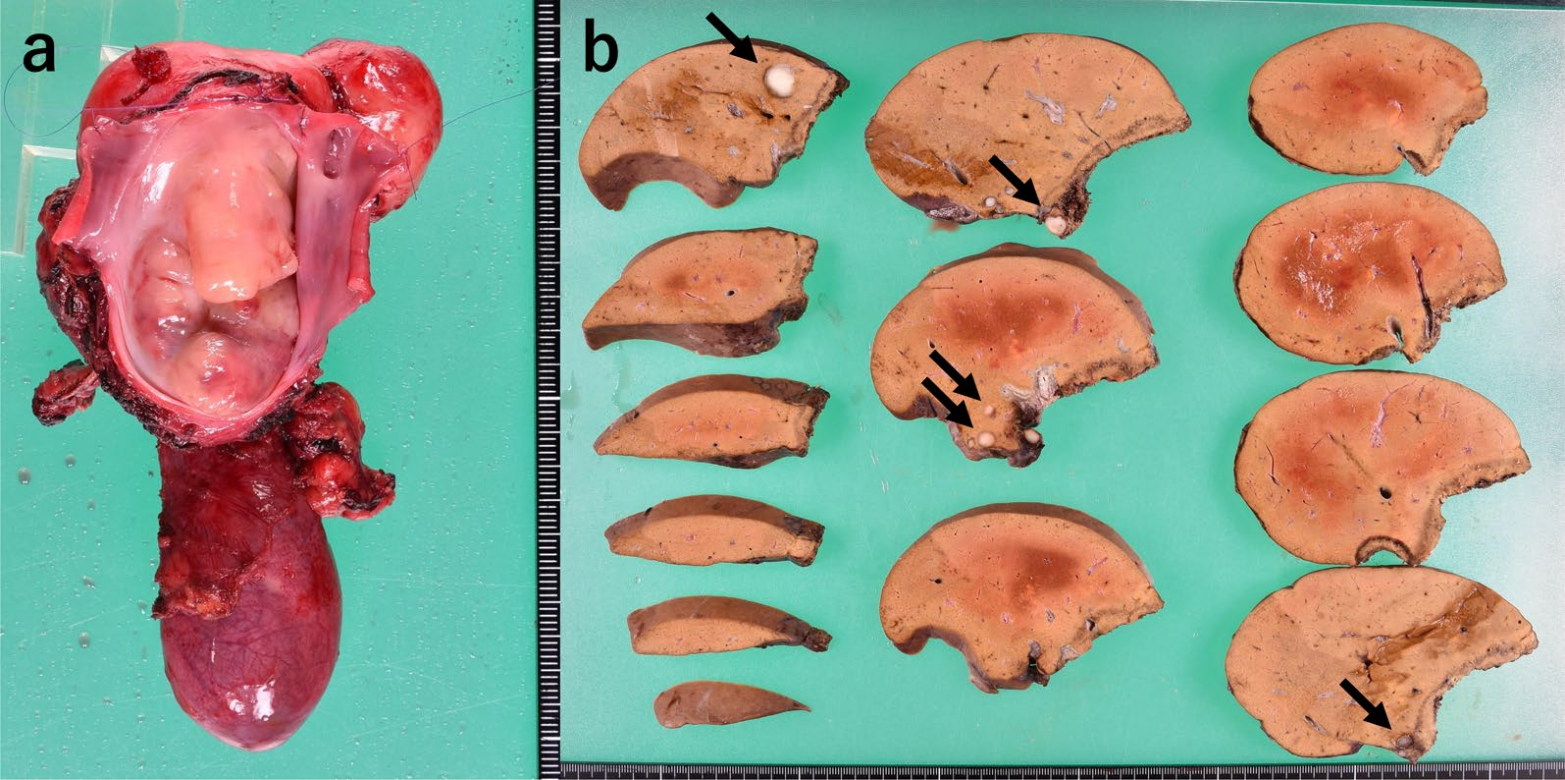
**a** Lumen of the removed IVC was occupied by a white segmental solid tumor. **b** There are several round white enriched lesions at S1 (10 mm × 1, 5 mm × 2, 2 mm × 1), S5 (15 mm × 1), and S6 (7 mm × 1) of the resected liver. A tumor in S1 was observed near the resection surface but the surgical margin was 1 mm. *IVC* inferior vena cava

**Fig. 4:**
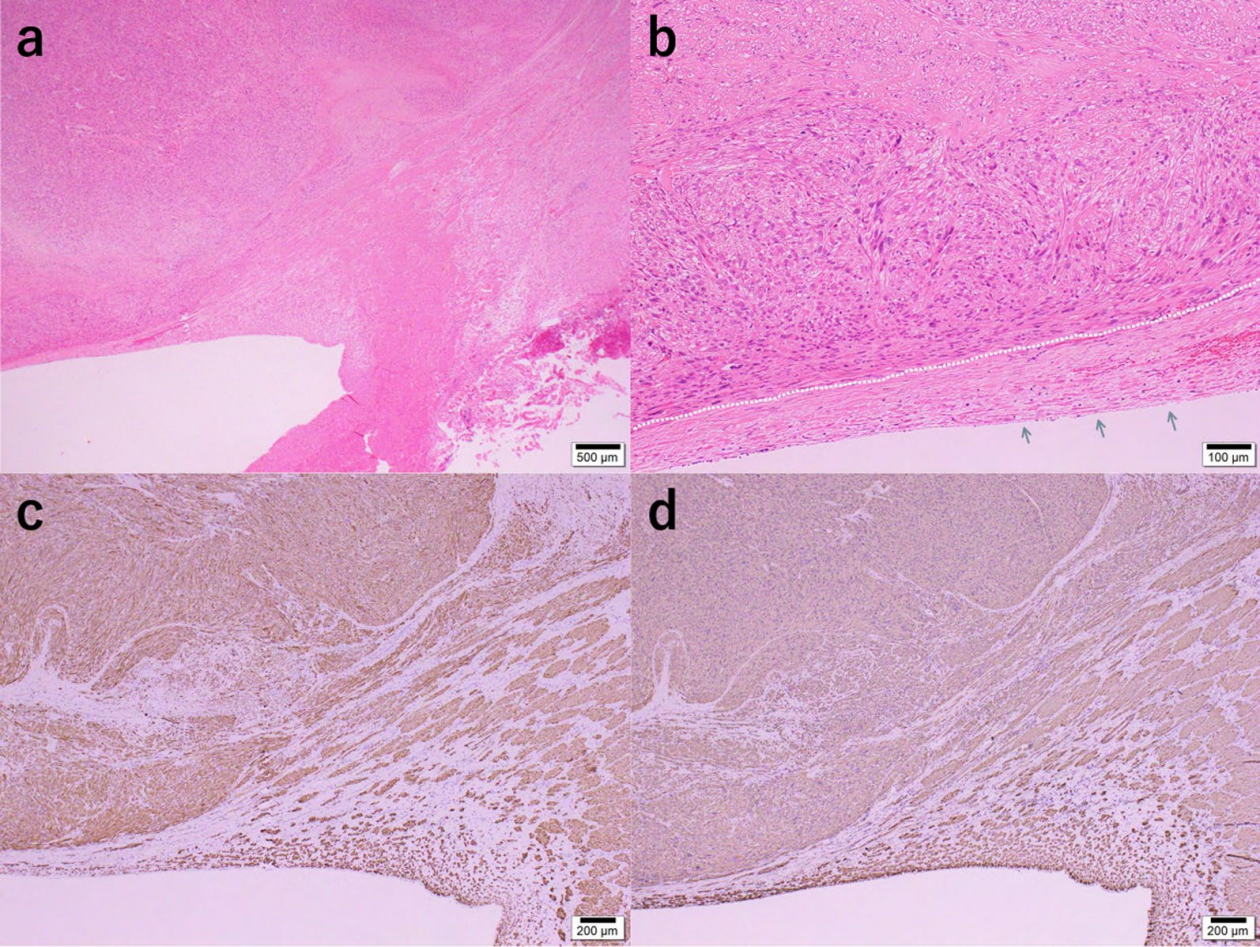
**a**, **b** Low-power (20×) image (**a**) and a high-power (100×) image (**b**) of the IVC lesion; spindle-shaped atypical cells are densely proliferated in a bundle-like structure. Polygonal and multinucleated atypical cells are also seen. Inside the white dotted line: tumor; arrow: IVC. **c**, **d** Immunohistochemistry: atypical cells were desmin-positive (**c**) and αSMA-positive (**d**). *IVC* inferior vena cava, *αSMA* α-smooth muscle actin

The lumen of the removed IVC was occupied by solid white lesions. The excised liver also contained a round, white, solid mass, similar to the primary lesion, and a 5 mm lesion was also identified near the detached surface of S1. However, its boundaries were clear and the transection surface was not expressed (Fig. 3). Pathological examination revealed spindle-shaped atypical cells densely proliferated in a bundle-like structure, and polygonal and multinucleated atypical cells were also observed. On immunostaining, the atypical cells were desmin-positive and α-smooth muscle actin (αSMA)-positive and were diagnosed as leiomyosarcoma (Fig. 4). Microscopic margins of the liver transection section were confirmed (R0), but there were many microscopic nodules scattered in the liver that were not noted preoperatively.

The patient’s postoperative course was uneventful; she was discharged on postoperative day 11. However, a CT and MRI performed on postoperative day 51 revealed distant metastatic recurrences in the remaining liver and right lung. The patient was immediately started on doxorubicin 60 mg/m^2^ and ifosfamide 2500 mg/m^2^ at a nearby pediatric specialty center. However, the metastatic lesion increased in size after 1 month. Pazopanib 550 mg/m^2^ was added to the then-current chemotherapy regimen but was ineffective. The patient was then switched to eribulin 1.4 mg/m^2^ and then to trabectedin 1.2 mg/m^2^. Trabectedin had the strongest antitumor effect, but also caused severe adverse effects. The patient passed away 19 months postoperatively due to uncontrollable hyperkalemia and intra-abdominal bleeding.

## Discussion

Previous reports on pediatric leiomyosarcomas can be broadly divided into two categories: Epstein–Barr virus (EBV)-related leiomyosarcomas in immunocompromised patients and others[20]. There have been several reports of leiomyosarcoma in non-immunocompromised patients; however, the only reports of primary vascular leiomyosarcomas such as the one in this case are of a 3-year-old girl with a lesion in the femoral vein and a 2-year-old girl with a lesion in the greater saphenous vein [6, 7]. Therefore, the information is very limited, and we referred to evidence in adult cases to determine the treatment plan.

In pediatric patients, tumor resection with replacement of the IVC is uncommon, and there have been only a few reports on neuroblastoma and other diseases [21]. On the other hand, there have been reports on adult patients with primary leiomyosarcoma of the IVC, where radical tumor resection was associated with 5- and 10-year survival rates, and of patients who underwent IVC replacement for vena cava tumors, including leiomyosarcoma, having improved outcomes [18, 22]. Preoperative chemotherapy has also been attempted, but there is no consensus on its efficacy [23–25]. Unfortunately, our patient had multiple liver metastases at the time of surgery, but since the metastases were confined to the right lobe, we thought that R0 resection could be performed through resection of the right lobe of the liver and replacement of the IVC. The liver is the major metastatic site of leiomyosarcoma, but there are few reports of cases with multiple liver metastases at the time of initial diagnosis (Table 1).

**Table 1:** Cases of multiple liver metastases in IVC leiomyosarcoma

Case	Author	Years	Age (year)	Sex	Location of liver metastases	Operations	Adjuvant chemotherapy	Recurrence (Months after surgery)	Non-surgical treatments	Prognosis (Months after Diagnosis)
1	Hardwigsen [26]	2005	54	Female	Right lobe	Right hepatectomy IVC resection	No	Yes (9 Months)	Unknown	Deceased (33 Months)
2	Kieffer [14]	2006	Unknown	Unknown	Unknown	No	–	–	None	Deceased (6 Months)
3	Motodaca [27]	2019	42	Male	Right lobe	Partial hepatectomy IVC reconstruction with PTFE graft	–	–	Chemotherapy	Alive (10 Months)
4	Sephien [28]	2019	46	Female	Both lobes	No	–	–	Chemotherapy	Deceased (29 Months)
5	Devi [29]	2022	24	Male	Both lobes	No	–	–	None	Deceased (1 Month)
6	Present case	2023	15	Female	Right lobe	Right hepatectomy IVC reconstruction with PTFE graft	No	Yes (2 Months)	Chemotherapy (After recurrence)	Deceased (19 Months)

There have been two cases in which surgery was performed for multiple liver metastases. In Case 1, tumor resection and right hepatectomy were performed as in the present case, but the patient had recurrence 9 months after surgery and passed away 33 months after surgery (the postoperative treatment after recurrence was unknown). In Case 3, the surgeons were unable to resect the entirety of the liver metastases, because numerous liver metastases were found during surgery. As a result, chemotherapy was administered postoperatively, and the patient was alive 10 months after the surgery. In our case, microscopic margins were secured; however, small tumors were scattered throughout the removed liver. Minute distant metastases that could not be seen on the preoperative CT and MRI scans could have been present in the liver and lungs at the time of surgery. This might have led to distant recurrence as early as 2 months after surgery. The benefit of adjuvant chemotherapy after R0 resection is controversial and it was not performed in Case 1. Since chemotherapy alone had a survival of 29 months (Case 4), postoperative chemotherapy may have been appropriate in our case, considering the possibility of residual lesions that could not be detected preoperatively.

Regarding chemotherapy for soft tissue tumors (including leiomyosarcoma), there is a meta-analysis showing a slight survival improvement with doxorubicin-based multidrug therapy; however, there are also reports suggesting that chemotherapy does not improve survival or recurrence risk [30–32]. In Japan when anthracycline chemotherapy fails, recently approved agents such as pazopanib, eribulin, and trabectedin are used as second-line or subsequent chemotherapy. Trabectedin was the most effective drug in this case but could not be administered on schedule due to hepatotoxicity. A study of the Japanese Musculoskeletal Oncology Group also showed that 96% of patients experienced adverse events and hepatotoxicity being the most frequent, at 37.9% of patients having grade 3 or higher hepatotoxicity. Additionally, the low acceptability of trabectedin is an issue [33].

It is a controversial question as to which molecularly targeted drug is the best option as second-line therapy. A randomized phase II trial of pazopanib, eribulin, and trabectedin in metastatic or unresectable advanced soft tissue sarcomas that have progressed after the initial chemotherapy including doxorubicin is currently underway in Japan, and the outcomes will be followed with interest.

## Conclusions

We report a rare case of primary vascular leiomyosarcoma in a pediatric patient. IVC resection and reconstruction combined with right hepatectomy were able to be safely performed even in a pediatric case. Although pazopanib, eribulin, and trabectedin are expected to play a role in the treatment strategy of leiomyosarcoma in the future, surgical resection remains the only curative option. However, since a high rate of hematogenous distant metastasis occurs, effective (preoperative) chemotherapy, including molecularly targeted drugs, should be established early and effectively combined with surgical treatment.

## Data Availability

Not applicable.

## Abbreviations

IVC: Inferior vena cava
CT: Computed tomography
MRI: Magnetic resonance imaging
GB: Gall bladder
RPV: Right portal vein
LRV: Left renal vein
PET: Positron emission tomography
e-PTFE: Expanded polytetrafluoroethylene
αSMA: α-Smooth muscle actin
CK: Creatinine kinase
EBV: Epstein–Barr virus
